# Fetal Brain-Sparing, Postnatal Cerebral Oxygenation, and Neurodevelopment at 4 Years of Age Following Fetal Growth Restriction

**DOI:** 10.3389/fped.2020.00225

**Published:** 2020-05-06

**Authors:** Anne E. Richter, Sahar Salavati, Elisabeth M. W. Kooi, Anne E. den Heijer, Anne B. Foreman, Mirthe H. Schoots, Caterina M. Bilardo, Sicco A. Scherjon, Jozien C. Tanis, Arend F. Bos

**Affiliations:** ^1^University of Groningen, University Medical Center Groningen, Beatrix Children's Hospital, Division of Neonatology, Groningen, Netherlands; ^2^University of Groningen, University Medical Center Groningen, Department of Obstetrics and Gynecology, Groningen, Netherlands; ^3^University of Groningen, University Medical Center Groningen, Department of Pathology and Medical Biology, Groningen, Netherlands; ^4^Department of Obstetrics and Gynecology, Amsterdam University Medical Center, VU University Medical Center, Amsterdam, Netherlands

**Keywords:** fetal doppler, cerebroplacental ratio, fetal brain-sparing, near-infrared spectroscopy, regional cerebral oxygen saturation, intelligence, behavior, executive function

## Abstract

**Objectives:** To assess the role of fetal brain-sparing and postnatal cerebral oxygen saturation (r_c_SO_2_) as determinants of long-term neurodevelopmental outcome following fetal growth restriction (FGR).

**Methods:** This was a prospective follow-up study of an FGR cohort of 41 children. Prenatally, the presence of fetal brain-sparing (cerebroplacental ratio < 1) was assessed by Doppler ultrasound. During the first two days after birth, r_c_SO_2_ was measured with near-infrared spectroscopy. At 4 years of age, intelligence (IQ points), behavior (T-scores), and executive function (T-scores) were assessed using the Wechsler Preschool and Primary Scale of Intelligence, Child Behavior Checklist, and Behavior Rating Inventory of Executive Function—Preschool Version, respectively. Using linear regression analyses, we tested the association (*p* < 0.05) between brain-sparing/r_c_SO_2_ and normed neurodevelopmental scores.

**Results:** Twenty-six children (gestational age ranging from 28.0 to 39.9 weeks) participated in the follow-up at a median age of 4.3 (range: 3.6 to 4.4) years. Autism spectrum disorder was reported in three children (11.5%). Fetal brain-sparing was associated with better total and externalizing behavior (betas: −0.519 and −0.494, respectively). R_c_SO_2_ levels above the lowest quartile, particularly on postnatal day 2 (≥ 77%), were associated with better total and internalizing behavior and executive functioning (betas: −0.582, −0.489, and −0.467, respectively), but also lower performance IQ (beta: −0.530). Brain-sparing mediated some but not all of these associations.

**Conclusions:** In this FGR cohort, fetal brain-sparing and high postnatal r_c_SO_2_ were—independently, but also as a reflection of the same mechanism—associated with better behavior and executive function. Postnatal cerebral hyperoxia, however, was negatively associated with brain functions responsible for performance IQ.

## Introduction

Fetal growth restriction (FGR) has been associated with altered brain structure and adverse neurodevelopmental outcome (1–3). In addition to an increased risk for preterm delivery, which poses a risk on neurodevelopmental outcome itself, FGR fetuses experience hemodynamic redistribution of their cardiac output (4, 5). Although this redistribution with preferential perfusion of the brain (brain-sparing) can be considered a protective compensatory response to placental insufficiency, it is also a sign of fetal compromise (6, 7). Numerous studies have associated brain-sparing with an increased risk of adverse perinatal outcome in both early and late onset FGR (8, 9). However, whether fetal brain-sparing is also associated with long-term neurodevelopmental delay is still under debate (10–13).

In addition to circulatory compromise *in utero*, FGR infants are susceptible to postnatal hemodynamic instability (14, 15). Organ immaturity associated with intrauterine nutrient deficiency and preterm birth, patent ductus arteriosus (PDA), maternal medication, and inotropic therapy interfere with adequate cerebral tissue oxygenation (16–18). Moreover, brain-sparing has been associated with impaired cerebral autoregulation, predisposing to fluctuations in blood flow and oxygenation (19). Evidence suggests that both postnatal cerebral hypo- and hyperoxia are associated with brain injury and neurodevelopmental delay (20, 21).

Despite intensive research in this field, studies evaluating fetal brain-sparing and/or postnatal cerebral oxygen saturation in relation to long-term neurodevelopmental outcome in FGR fetuses are scarce. Moreover, the extent to which both contribute to neurodevelopmental outcome following FGR has not been studied yet. We therefore aimed to longitudinally explore the effect of both fetal brain-sparing and postnatal cerebral oxygen saturation (r_c_SO_2_) on intelligence, behavior, and executive functioning (EF) in 4-year-old children with fetal growth restriction. We hypothesized that fetal brain-sparing and postnatal cerebral hypo- but also hyperoxia independently and cumulatively contribute to neurodevelopmental delay.

## Materials and Methods

### Study Design and Population

This was a prospective follow-up study of an FGR cohort born between June 2012 and May 2014 in the University Medical Center Groningen (UMCG), The Netherlands. All children in this cohort were recruited antenatally, based on FGR defined as a fetal abdominal circumference or estimated fetal weight below the 10th percentile or a deflecting fetal growth curve by more than 30 percentiles compared with the previous examination. Exclusion criteria were structural or chromosomal abnormalities, multiple pregnancy, or evidence of intrauterine infection. Surviving infants with available fetal Doppler and/or neonatal r_c_SO_2_ measurements on the first two days after birth were eligible for follow-up at 4 years of age, if consent for follow-up was given at prenatal inclusion. Children insufficiently mastering the Dutch language due to upbringing with another language were excluded from intelligence testing as this may negatively affect test results. The study was approved by the Institutional Ethics Committee and written informed consent was obtained in all cases.

### Fetal and Neonatal Measurements

Upon diagnosis of FGR, fetal hemodynamic parameters were measured at least once a week (twice upon admission) by Doppler sonography, including the pulsatility index (PI) of the umbilical artery (UA), the middle cerebral artery (MCA), and the ductus venosus (DV). The cerebroplacental ratio (CPR) was calculated by dividing the PI of the MCA by that of the UA. A CPR <1 was defined as fetal brain-sparing (22). An abnormal flow in the DV was defined as a PI > 95th percentile or an absent or reversed a-wave. The last measurement before birth was used for analysis.

On day 1 and 2 after birth, we measured the r_c_SO_2_ with near-infrared spectroscopy using the INVOS 5100C device and the neonatal OxyAlert Sensor (Medtronic, Dublin, Ireland). The sensor was placed on the right or left frontoparietal side of the head for a minimum of two hours per day. Data were retrieved at five-second intervals.

### Neurodevelopmental Follow-up at 4 Years

At 4 years of age, the Wechsler Preschool and Primary Scale of Intelligence for children aged 4 to 7 years (WPPSI, 3rd edition) was performed. The following core subtests were tested to retrieve the full scale intelligence quotient (FSIQ), verbal IQ (VIQ), and performance IQ (PIQ): block design, information, matrix reasoning, vocabulary, picture concepts, word reasoning, and coding (23). If a child had previously been tested, we asked permission to use these test results as repetition can improve outcome and introduce bias. An IQ score <85 (one standard deviation below the mean) was defined below average. Failure to derive an IQ score due to inadequate responses was treated as missing data.

To assess behavior and EF, two parent-reported questionnaires were applied: the Child Behavior Checklist (CBCL) for ages 1.5–5 years and the Behavior Rating Inventory of Executive Function—Preschool Version (BRIEF-P) for children aged 2–5 years. The CBCL comprised questions regarding internalizing behavior (emotional reactivity, anxiety/depression, somatic complaints, and withdrawal), externalizing behavior (attention and aggressive behavior), and sleep problems, which together constituted a total behavior scale (24). The BRIEF-P allowed for calculation of the Inhibitory Self-Control Index (ISCI, i.e., the child's ability to adjust its behavior using appropriate inhibitory self-control), the Flexibility Index (FI, i.e., the child's capacity to adapt to change), the Emergent Metacognition Index (EMI, i.e., the child's capacity to effectively solve problems using working memory and planning), and a total EF score comprising all three indices (25). For both CBCL and BRIEF-P normed T-scores were calculated. Abnormal scores were defined as T-scores ≥ 60 and ≥ 65, respectively.

### Patient Characteristics

Available perinatal data with potential influence on fetal brain-sparing, postnatal cerebral oxygenation, and neurodevelopment, such as maternal smoking, placental histology, gestational age (GA) at birth, birth weight (z-scores), head circumference at birth (z-score), Apgar score at 5 min, arterial cord blood pH and base excess, the need for mechanical ventilation, a hemodynamically significant PDA, necrotizing enterocolitis, sepsis, and intracranial pathology were collected. At the age of 4 years, length, weight, and head circumference were measured and any sensory problems (visual acuity, hearing) were recorded. Additionally, we retrieved information on maternal socioeconomic status (based on educational background).

### Sample Size Calculation

In a previous study, we reported that 39% of these FGR infants showed evidence of fetal brain-sparing, which was in turn significantly related to a higher postnatal cerebral oxygen saturation and abnormal general movements (GMs) at 1 week after birth (26, 27). Because the quality of GMs is closely related to IQ, which was found to be 15 points lower (i.e., 1 SD) by Bruggink et al. if GMs were abnormal, we expected to find a 1 SD difference of IQ between children with and without fetal brain-sparing (28). For the individual child, this is a highly relevant difference. With a power of 80%, an alpha of 5%, and an experiment-to-control subjects ratio of 0.6, at least 21 children needed to be included in the follow-up to reach statistical significance.

### Statistical Analysis

The statistical software package SPSS 23.0 (IBM Corporation, Armonk, New York, USA) was used for analyses. First, we explored the association between r_c_SO_2_ and the continuous neurodevelopmental outcome scales using Spearman's rank correlation analysis and scatterplots. Graphical data suggested a linear relationship with a potential r_c_SO_2_ threshold for abnormal outcome scores between the first (lowest) and the second quartile (cut-off value of 72% for day 1 and 77% for day 2). Second, we performed separate linear regression analyses to examine the effect of (1) fetal brain-sparing and (2) postnatal r_c_SO_2_ above the lowest quartile on the continuous neurodevelopmental outcome scales. Patient characteristics associated with both outcome and either fetal brain-sparing or postnatal r_c_SO_2_ (*p* < 0.1 using Chi square, Mann-Whitney *U, t*-test, or Spearman's correlation) were regarded as confounders and adjusted for by adding them to the model. Third, we tested whether r_c_SO_2_ was related to brain-sparing using Mann-Whitney *U* test. If so, brain-sparing was entered into the linear regression models for r_c_SO_2_ to explore mediation. For regression analyses, a *p*-value below 0.05 was considered significant.

## Results

**Figure 1:**
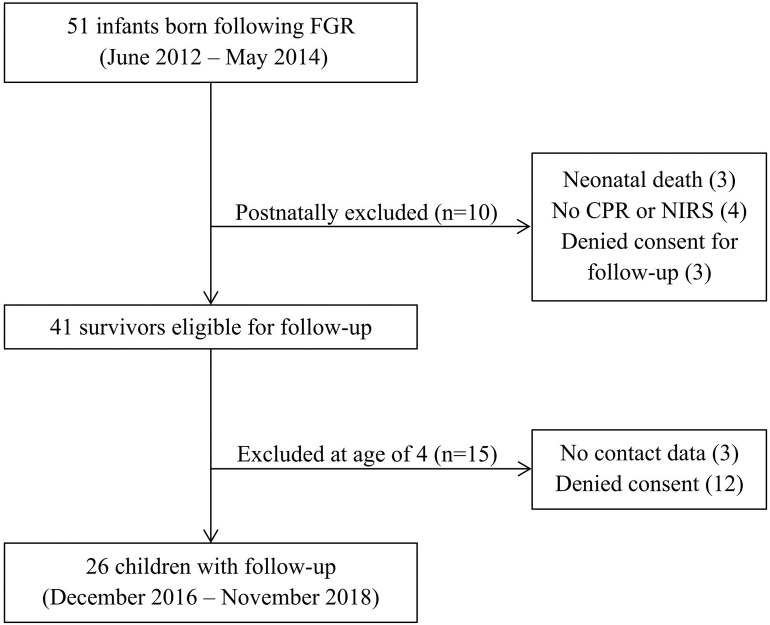
Inclusion flowchart. CPR, cerebroplacental ratio; FGR, fetal growth restriction; NIRS, near-infrared spectroscopy.

Out of 51 FGR infants, 48 survived the neonatal period and 41 were eligible for follow-up based on consent and perinatal measurements. At 4 years, three children were lost to follow-up due to lack of contact information or response, and the parents of 12 children withdrew consent to follow-up. Non-participants had a median GA of 34.4 [interquartile range (IQR) 29.3–38.4] weeks and a median birth weight z-score of −1.28 [IQR −1.66–−0.70]. Eventually, 26 children participated in neurodevelopmental testing, conducted from December 2016 to November 2018. A detailed inclusion flowchart is depicted in Figure 1. Perinatal patient characteristics of children declining or lost to follow-up, such as gestational age, birth weight (z-score), head circumference (z-score), CPR (z-score), postnatal cerebral oxygen saturation, neonatal or gestational complications, were not significantly different from those of participating children (data not shown).

### Characteristics of Study Participants

Detailed perinatal and childhood characteristics are given in Table 1. Seven infants (27%) were born very preterm (i.e., <32 weeks GA). Seventeen infants (65%) had been admitted to neonatal intensive care and nine infants (35%) required mechanical ventilation. 22 children had an arterial cord blood pH < 7.35, but none had an arterial cord blood pH < 7.00 and/or base excess < −12. Routine postnatal ultrasounds did not reveal major cerebral pathologies in any neonate.

**Table 1:** Cohort characteristics (*n* = 26, if not indicated otherwise).

	***n*** **(%) or median [range]**	
**Maternal characteristics**		
Smoking during pregnancy	7 (27)	
Preeclampsia	4 (15)	
PPROM	2 (8)	
Socioeconomic status		
Low	—-	
Middle	14 (54)	
High	11 (42)	
**Perinatal infant characteristics**		
Female	12 (46)	
Abnormal flow UA (PI >95th percentile or absent/reversed flow)	18 (69)	
Abnormal flow MCA (PI <5th percentile), measured in *n* = 25	8 (31)	
Fetal brain-sparing (CPR <1), measured in *n* = 25	11 (44)	
Abnormal flow in the DV (PI >95th percentile/absent or reversed a-wave), measured in *n* = 23	13 (56)	
Cesarean section	19 (73)	
Gestational age, weeks	35.1 [28.0; 39.9]	
GA <32 weeks	7 (27)	
Birth weight, z-score	−2.7 [−5.9; −0.29]	
Small-for-gestational age	23 (89)	
Head circumference, z-score	−2.1 [−4.3; −0.4]	
Apgar score at 5 min	8.5 [4; 10]	
Arterial cord blood pH	7.26 [7.92; 7.41]	
Arterial cord blood BE (mmol/l)	−6 [−11; −1]	
Admission to NICU	17 (65)	
Mechanical ventilation	9 (35)	
Hemodynamically significant PDA	2 (8)	
Neonatal sepsis	1 (4)	
IVH/PVL	–	
Transient PVE	5 (19)	
**Developmental characteristics at 4 years**		
Height, z-score	−0.45 [−2.19; 3.40]	
Weight, z-score	−0.93 [−2.68; 4.37]	
Head circumference, z-score	−0.57 [−3.73; 2.09]	
Suspected or diagnosed ASD	3 (12)	
**Cognitive outcome at 4 years**	**Median [range]**	**Below average (IQ** ** <85)**
Full Scale IQ (*n* = 19)	94 [63; 123]	4 (21)
Verbal IQ (*n* = 19)	97 [71; 120]	4 (21)
Performance IQ (*n* = 20)	94 [72; 120]	3 (15)
**Behavioral outcome at 4 years (T-score)**	**Median [range]**	**Abnormal (T-score** **≥60)**
Total behavior (*n* = 25)	56 [28; 72]	8 (32)
Internalizing behavior (*n* = 25)	55 [29; 73]	8 (32)
Externalizing behavior (*n* = 25)	56 [28; 68]	7 (28)
**Executive function at 4 years (T-score)**	**Median [range]**	**Abnormal (T-score** **≥65)**
Total executive function (*n* = 24)	60 [34; 76]	9 (38)
Inhibitory Self-Control Index (*n* = 25)	57 [35; 77]	8 (32)
Flexibility index (*n* = 25)	58 [37; 95]	6 (24)
Emergent metacognition index (*n* = 24)	55 [36; 71]	7 (29)

*Percentages were calculated based on number of total measurements, which may deviate from the number of included infants in this cohort as indicated. ASD, autism spectrum disorder (as reported by parents); a-wave, atrial contraction wave; BE, base excess; CPR, cerebroplacental ratio; DV, ductus venosus; GA, gestational age; IQ, intelligence quotient; IVH, intraventricular hemorrhage; MCA, middle cerebral artery; NICU, neonatal intensive care unit; PDA, patent ductus arteriosus; PI, pulsatility index; PPROM, prolonged premature rupture of membranes (>12 h); PVE; periventricular echodensities; PVL, periventricular leukomalacia; UA, umbilical artery*.

The median age at follow-up was 4.3 (total range 3.6–4.4) years. No major health or neurosensory problems were recorded, but three children were parentally reported to be diagnosed with or highly suspected of autism spectrum disorder (ASD). One of them did not participate in the WPPSI and one had been tested by an external institution at the age of 3.6 years, whose results we were allowed to use. Another child did not participate in the WPPSI but only questionnaires, as the mother judged the infant not emotionally apt. In four children (15%, including the third case of ASD), the WPPSI was largely unsuccessful (one resulting in only a valid PIQ but not VIQ/FSIQ score) due to severe concentration and/or behavioral problems or because tasks were not understood or reacted to appropriately. None of the four children showed fetal brain-sparing and three had repeatedly low r_c_SO_2_ levels (56–75%) on both postnatal days.

### Fetal Brain-Sparing and Neurodevelopmental Outcome at 4 Years

In 25 infants (96%) the cerebroplacental ratio was available. In 11 infants (44%) fetal brain-sparing was present during last prenatal ultrasound. Among the infants without brain-sparing, seven versus nine infants demonstrated abnormal UA flow during last versus earlier prenatal ultrasound examinations. At least two infants without brain-sparing but abnormal UA flow during last prenatal ultrasound demonstrated brain-sparing during earlier ultrasound examinations. Their r_c_SO_2_ levels were 66% and 71% on day 1 and 64% and 89% on day 2, respectively. Placental histology was not significantly different between children with and without brain-sparing, except for a tendency toward a lower placental weight percentile in those with fetal brain-sparing (Table 2).

**Table 2:** The presence or absence of fetal brain-sparing (CPR <1) at last prenatal ultrasound in relation to placental histology, detailed fetal Doppler indices, and perinatal outcome.

	**Fetal brain-sparing *n* = 11**	**No fetal brain-sparing *n* = 14**	***p*-value**
**Placental histology^†^**			
Maternal vascular underperfusion	5 (50)	6 (50)	1.000
Fetal thrombotic vasculopathy	4 (40)	3 (25)	0.452
Ascending intrauterine infection	2 (20)	2 (17)	0.840
Chronic deciduitis	4 (40)	4 (33)	0.746
Villitis of unknown etiology	2 (20)	1 (8)	0.427
Increase in nucleated RBCs	3 (30)	2 (17)	0.457
Placental weight (gram)	262 [206; 436]	321 [149; 507]	0.228
<10th percentile	9 (90)	7 (58)	0.097*
**Doppler characteristics at last prenatal ultrasound**			
Abnormal flow UA (PI > 95th percentile or absent/reversed flow)	11 (100)	7 (50)	0.006**
Abnormal flow MCA (PI <5th percentile)	7 (64)	1 (7)	0.003**
Abnormal flow DV (PI > 95th percentile/absent or reversed a-wave)	7 (64)	6 (43)	0.510
**Perinatal outcome**			
Cesarean delivery	11 (100)	8 (57)	0.013**
Gestational age, weeks	32.1 [29.1; 37.6]	36.2 [28.0; 39.9]	0.085*
<32 weeks	5 (46)	2 (14)	0.085*
Birth weight, z-score	−3.42 [−5.36; −1.79]	−2.44 [−5.87; −0.29]	0.066*
Head circumference, z-score	−1.86 [−4.31; −0.43]	−2.12 [−3.62; −0.92]	0.687
Arterial cord blood pH	7.21 [7.02; 7.31]	7.28 [7.16; 7.41]	0.075*
Arterial cord blood BE (mmol/l)	−7 [−11; −2]	−6 [−10; −1]	0.515

*Data are given as medians [range] or numbers (%)*.

^**^ and ^*^ present a difference between groups below the 5% and 10% significance level, respectively.

† *as examined in n = 10 (fetal brain-sparing present) and n = 12 (fetal brain-sparing absent) placentas by a perinatal pathologist according to the criteria applicable at the time of examination (46–57). BE, base excess; CPR, cerebroplacental ratio; DV, ductus venosus; MCA, middle cerebral artery; UA, umbilical artery; PI, pulsatility index; RBC, red blood cell*.

Children with fetal brain-sparing during last ultrasound had a lower median GA and were slightly but not significantly smaller and more acidotic at birth than children without brain-sparing (Table 2). There was no difference in neonatal complications. At the age of 4, one child with and two without fetal brain-sparing (but abnormal UA flow) during last ultrasound were diagnosed with ASD.

**Figure 2:**
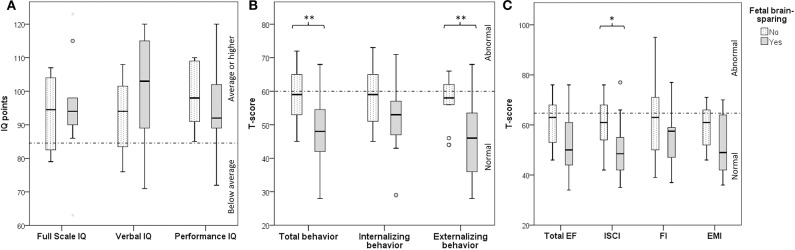
Box -Whisker plots depicting differences in neurodevelopmental outcome (**A**: cognition, **B**: behavior, **C**: executive function) in 4-year-old children with and without fetal brain-sparing following fetal growth restriction. Boxes represent interquartile ranges, whiskers the total range of values excluding outliers, circles represent outliers and asterisks extreme outliers. Dotted lines indicate the cut-off between normal and abnormal scores. *and **demonstrate a difference below the 10 and 5% significance level, respectively. EF, executive function; EMI, Emergent Metacognition Index; FI, Flexibility Index; IQ, intelligence quotient; ISCI, Inhibitory Self-Control Index.

Figure 2 depicts the association between brain-sparing and neurodevelopmental outcomes. Fetal brain-sparing was not associated with IQ (Table 3). It was, however, associated with better total behavior (i.e., a T-score of 11 points less than with absence of brain-sparing) and better externalizing behavior. Infants with fetal brain-sparing also tended to have better inhibitory self-control. If indicated, the association was adjusted for gestational age, which positively correlated with the T-scores for total behavior (Spearman's rho = 0.421, *p* = 0.036), total EF (rho = 0.410, *p* = 0.046), and EMI (rho = 0.480, *p* = 0.018).

**Table 3:** The association between fetal brain-sparing or the lowest quartile of cerebral tissue oxygen saturation on day 1 and 2 after birth and neurodevelopmental outcome at 4 years of age in children born following fetal growth restriction, using separate linear regression models.

	**Cognition**			**Behavior**			**Executive function**			
	***FSIQ***	***VIQ***	***PIQ***	***Total***	***Internalizing***	***Externalizing***	***Total***	***ISCI***	***FI***	***EMI***
**Fetal brain-sparing**										
B [95%CI]	2.0 [−12.3;16.3]	7.8 [−5.9;21.4]	−5.5 [−17.5;6.5]	−11.3^†^ [−20.0;−2.6]	−6.3 [−14.9;2.3]	−11.3 [−20.0;−2.5]	−8.0^†^ [−18.9;2.9]	−9.1 [−19.0;0.9]	−7.7 [−19.8;4.3]	−6.2^†^ [−15.3;3.0]
Beta	0.074	0.287	−0.227	−0.519	−0.309	−0.494	−0.336	−0.374	−0.273	−0.302
*p*–value	0.771	0.248	0.350	0.013**	0.142	0.014**	0.140	0.072*	0.197	0.176
R^2^	0.5%	8.3%	5.2%	28.6%	9.5%	24.4%	18.6%	14.0%	7.5%	21.0%
**r**_**c**_**SO**_**2**_ **≥** **72% day 1**										
B [95%CI]	4.4 ^§^ [−12.9;21.7]	9.2 ^§^ [−9.5;27.9]	1.7 ^§^ [−14.5;17.9]	−10.8 [−20.5;−1.2]	−9.1 [−18.4;0.3]	−7.6 [−18.1;3.0]	−10.6 [−22.6;1.5]	−9.5 [−20.9;1.9]	−13.4 [−26.0;0.9]	−9.8 [−20.1;0.6]
Beta	0.133	0.256	0.060	−0.444	−0.395	−0.302	−0.362	−0.338	−0.420	−0.385
*p*–value	0.593	0.313	0.828	0.030**	0.056*	0.152	0.082*	0.099*	0.037**	0.063*
R^2^	26.8%	25.6%	13.0%	19.7%	15.6%	9.1%	13.1%	11.4%	17.6%	14.9%
**r**_**c**_**SO**_**2**_ **≥** **77% day 2**										
B [95%CI]	−12.2 [−28.8;4.3]	−4.7 [−24.2;14.8]	−15.2 [−28.7;−1.8]	−14.8 [−25.0;−4.6]	−12.2 [−23.0;−1.4]	−11.1 [−22.6;0.4]	−14.4 [−28.0;−0.9]	−14.0 [−26.0;−1.9]	−19.1 [−32.8;−5.4]	−12.1 [−23.5;−0.6]
Beta	−0.390	−0.136	−0.530	−0.582	−0.489	−0.430	−0.467	−0.485	−0.557	−0.462
*p*–value	0.135	0.614	0.029**	0.007**	0.029**	0.059*	0.038**	0.026**	0.009**	0.040**
R^2^	15.2%	1.9%	28.1%	33.9%	23.9%	18.5%	21.8%	23.5%	31.0%	21.4%

† *The association was adjusted for gestational age*.

§ *The association was adjusted for head circumference at birth (z-score)*.

^**^ and ^*^ present an association below the 5% and 10% significance level, respectively. B, unstandardized coefficient; Beta, standardized coefficient; CI, confidence interval; EMI, Emergent Metacognition Index; FI, Flexibility Index; FSIQ, Full Scale Intelligence Quotient; ISCI, Inhibitory Self-Control Index ; PIQ, Performance Intelligence Quotient; r_c_SO_2_, regional cerebral tissue oxygen saturation; R^2^, Nagelkerke's R squared (percentage of variance in outcome explained by either brain-sparing or r_c_SO_2_ and adjusted confounders); VIQ, Verbal Intelligence Quotient.

Excluding the two cases with potential loss of brain-sparing (of which one was reported to have ASD) eliminated the trend association between brain-sparing and ISCI (*p* > 0.1) and reduced the strength of association between brain-sparing and better externalizing behavior (0.05 < *p* < 0.1, data not shown). Treating the two cases as brain-sparing, eliminated the association between brain-sparing, ISCI, and externalizing behavior, and reduced its strength of association with a better total behavior (0.05 < *p* < 0.1, data not shown).

To explore the impact of emerging fetal cardiac decompensation, we performed the same analyses with brain-sparing in combination with abnormal DV flow (*n* = 7). This was associated with a lower PIQ (B = −13.0, 95% CI = −24.5–−1.5, *p* = 0.029), better total behavior (B = −11.1, 95% CI = −20.4–−1.7, *p* = 0.023), and better inhibitory self-control (B = −11.9, 95% CI = −22.3–1.5, *p* = 0.026). There was also a tendency toward better externalizing behavior (B = −9.0, 95% CI = −19.3–1.3, *p* = 0.084) and total EF (B = −10.6, 95% CI = −21.2–0.03, *p* = 0.051). Adjustment for other variables was not indicated.

### Postnatal Cerebral Oxygen Saturation and Neurodevelopmental Outcome at 4 Years

Cerebral rSO_2_ was measured in 25 infants. Average r_c_SO_2_ ranged from 56 to 92% on day 1 (median 83%, IQR 71–89%) and from 64 to 94% on day 2 (median 84%, IQR 76–90%). It was not associated with GA at birth or birth weight, but r_c_SO_2_ on day 1 positively correlated with head circumference z-scores (*p* < 0.1). Among neonatal and maternal characteristics, only a PDA was significantly associated with lower r_c_SO_2_ on both days (*p* < 0.1).

**Figure 3:**
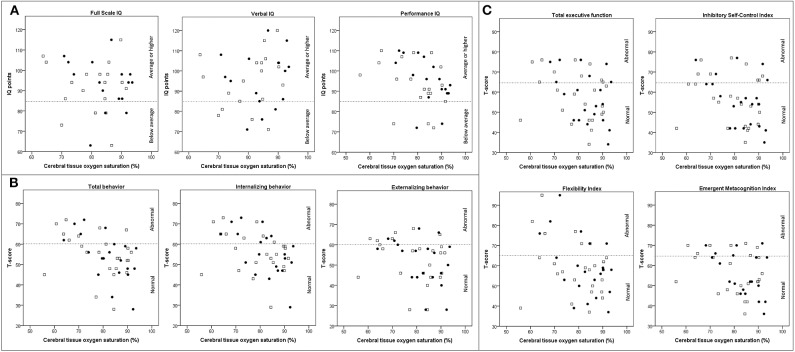
Scatterplots depicting the association between cerebral oxygen saturation on postnatal day 1 (white squares), day 2 (black dots), and neurodevelopmental outcome (**A**: cognition, **B**: behavior, and **C**: executive function) in children at 4 years of age following fetal growth restriction. Dotted lines indicate the cut-off between normal and abnormal scores. IQ, intelligence quotient.

Figure 3 depicts a linear relationship between r_c_SO_2_ and neurodevelopmental outcome. Correlation analyses confirmed that a higher r_c_SO_2_ on day 2 but not day 1 was associated with lower PIQ, but better total and internalizing behavior and a tendency toward better emotional flexibility and emergent metacognition (i.e., lower T-scores, Table 4). As scatterplots suggested a potential r_c_SO_2_ threshold toward abnormal IQ and T-scores at around 70–80% for both days, we build a binary variable using the cut-off values between the lowest and the second lowest quartile (72% on day 1 and 77% on day 2), which was entered into regression analyses. R_c_SO_2_ values equal to or above 77% on day 2 were associated with worse PIQ, but better total and internalizing behavior, and better EF (all domains, Table 3). There was also a tendency toward better externalizing behavior. Moreover, r_c_SO_2_ values equal to or above 72% on day 1 were associated with better total behavior and ability to adapt to change. There was also a tendency toward better internalizing behavior, total EF, inhibitory self-control, and emergent metacognition for values above the lowest quartile on day 1.

**Table 4:** The association between cerebral tissue oxygen saturation (r_c_SO_2_) on day 1 and 2 after birth and neurodevelopmental outcome at 4 years of age in children born following fetal growth restriction, using Spearman's rank correlation analyses.

	**r**_****c****_**SO**_****2****_ **day 1**		**r**_****c****_**SO**_****2****_ **day 2**	
	**Correlation coefficient**	***p*-value**	**Correlation coefficient**	***p*-value**
**Cognition (IQ)**				
Full-Scale	−0.031	0.902	−0.291	0.275
Verbal	0.236	0.345	0.172	0.524
Performance	−0.370	0.119	−0.603	0.010**
**Behavior (T-scores)**				
Total	−0.342	0.102	−0.554	0.011**
Internalizing	−0.267	0.207	−0.528	0.017**
Externalizing	−0.261	0.218	−0.362	0.117
**Executive function (T-scores)**				
Total	−0.216	0.310	−0.370	0.108
ISCI	−0.184	0.378	−0.355	0.114
FI	−0.226	0.277	−0.399	0.073*
EMI	−0.147	0.492	−0.427	0.060*

** and ^*^ present an association below the 5% and 10% significance level, respectively. EMI, Emergent Metacognition Index; FI, Flexibility Index; ISCI, Inhibitory Self-Control Index; IQ, Intelligence Quotient; r_c_SO_2_, regional cerebral tissue oxygen saturation.

**Figure 4:**
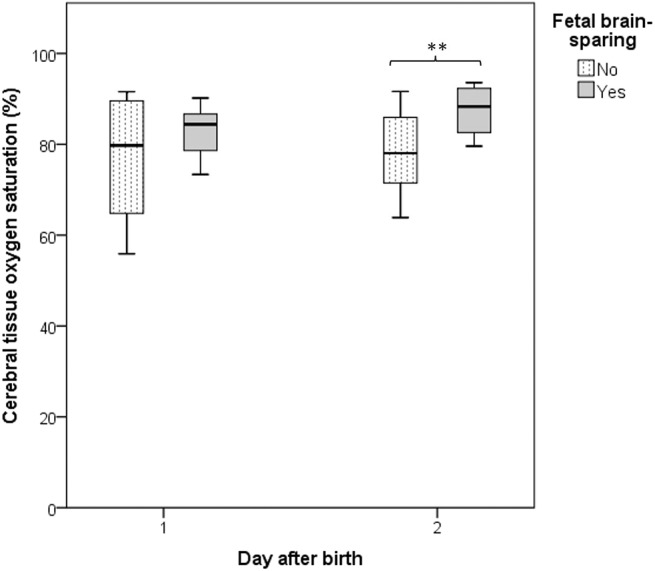
Box-Whisker plot depicting the association between fetal brain-sparing and the cerebral oxygen saturation on postnatal days 1 and 2 in infants born following fetal growth restriction. Boxes represent interquartile ranges and whiskers the total range of values. **demonstrates a difference below the 5% significance level.

Fetal brain-sparing was associated with higher r_c_SO_2_ on postnatal day 2 (*p* = 0.020, Figure 4). Forcing both into one regression model, the majority of associations disappeared or reduced in strength, further supporting mediation between the two variables. Only r_c_SO_2_ levels above the lowest quartile remained associated with better emotional flexibility (B = −18.8, 95% CI = −35.5–−2.5, *p* = 0.029) and a tendency toward better total and internalizing behavior (B = −11.6, 95% CI = −25.0–1.8, *p* = 0.084, and B = −12.2, 95% CI = −25.9–1.4, *p* = 0.077).

## Discussion

In this follow-up study of FGR infants, fetal brain-sparing was not associated with IQ at 4 years, but with better total and externalizing behavior. Similarly, postnatal r_c_SO_2_ above the lowest quartile on day 1 and 2 were associated with better total and internalizing behavior and executive functioning. However, the opposite was true for the association between PIQ and r_c_SO_2_ levels on postnatal day 2. Brain-sparing, which greatly influenced r_c_SO_2_ levels on day 2, seemed to mediate some but not all associations between r_c_SO_2_ and outcome.

Other studies have evaluated the effect of brain-sparing on long-term neurodevelopment in FGR children. Korkalainen et al. reported that, adjusted for GA, only abnormal umbilical or DV flow, but not a low CPR were associated with a need for physiotherapy, special education, or speech therapy at 9 years (29). Bellido-Gonzalez et al. reported that, in comparison with appropriate-for-GA (AGA) infants, late-onset FGR infants with a CPR below the 5th percentile had more cognitive deficits at 6–8 years than late-onset FGR infants with a normal CPR (30). Similarly, the TRUFFLE trial, evaluating whether DV waveforms may better guide delivery in early-onset FGR than short-term variation of fetal heart rate, reported a weak association between abnormal neurodevelopment at 2 years and higher umbilicocerebral ratios (more brain-sparing) at inclusion, but not at one week before delivery (31). They concluded that, in early FGR, brain-sparing is less useful to guide elective delivery (32). Moreover, despite its neuroprotective function, early onset and prolonged brain-sparing in FGR represent a risk factor for poor neurodevelopment.

To our knowledge, this is the first study limited to FGR children reporting equal and even better neurodevelopmental outcomes following brain-sparing, which was contrary to our hypothesis. In the same cohort, however, we previously reported brain-sparing to be associated with abnormal GMs one week after birth, but not at three months post-term (27). Literature suggests that only consistently abnormal GMs until eight weeks post-term are predictive of low IQ later in life (28). Thus, although associated with adverse perinatal outcome, brain-sparing in FGR seems to be beneficial and even critical for long-term neurodevelopment. Our data even suggest that brain-sparing outweighs any benefits a higher GA at birth may have for neurodevelopment, since FGR children without brain-sparing, born at a later GA, experienced more behavior and EF problems than more preterm babies with brain-sparing. However, our findings may also relate to onset of FGR relative to brain development. While the brain requires less oxygen earlier in gestation, the third trimester presents a period of increased brain growth and oxygen demand (33). Early and late onset FGR may therefore differentially affect the brain, as was recently demonstrated in fetal sheep (34). Moreover, the same study demonstrated that fetuses with late-onset FGR become hypoxic faster than fetuses with early-onset FGR. Thus, hypoxia during the last trimester may be more difficult to compensate and more harmful to the brain than at earlier stages, overriding any beneficial effects of advanced GA. Although a reduced CPR would frequently be the first sign of fetal hemodynamic adaptation in late FGR, severely compromised fetuses can show preterminal loss of compensatory cerebral vasoreactivity, which was possibly observed in at least two children without brain-sparing and may further explain poorer neurodevelopment in these patients (8, 35).

As expected, postnatal cerebral hypoxia negatively affected long-term behavior and EF following FGR. This included r_c_SO_2_ values below 72% (postnatal day 1) and 77% (postnatal day 2) as measured with the neonatal INVOS sensor. Verhagen et al. also report poorer cognition and motor function at 2–3 years following saturations below 72% on postnatal day 1 in preterm AGA infants (20). Interestingly, we observed the opposite for PIQ, which was poorer with saturations above 77% on day 2. Although Verhagen et al. similarly reported poorer cognition at r_c_SO_2_ levels above 83% on day 1, Verhagen et al. (20) the inverse relationship between r_c_SO_2_ and PIQ is puzzling. PIQ has, however, been related to motor function, and oxidative stress is known to cause white matter and motor neuron injury, in particular if preceded by hypoxia (36–41). Additionally, hemodynamic redistribution may occur within the brain from frontal regions to basal ganglia in FGR fetuses with brain-sparing and impending cardiac failure, as heralded by abnormal DV waveforms (42–44). As our data support an association between fetal decompensation and poorer PIQ, intracerebral perfusional redistribution may indeed contribute to a poorer PIQ in these children. Elective delivery based on DV waveforms in early FGR may therefore benefit neurodevelopment, as suggested by the TRUFFLE study (45). Moreover, brain-sparing was related to high postnatal r_c_SO_2_, suggesting that the proposed neuroprotective effects of postnatal r_c_SO_2_ are merely a reflection of preferential (intra)cerebral perfusion. Indeed, brain-sparing seemed to mediate some of the associations between r_c_SO_2_ and neurodevelopment, but not all. Postnatal events (second hit) influencing brain oxygenation may therefore also be important.

We acknowledge some limitations. First, a high loss to follow-up and a small sample reduced statistical power. Additionally, some children were unable to perform the WPPSI due to severe cognitive or behavioral problems, further decreasing the power to detect IQ differences. Of note, none of these infants showed fetal brain-sparing and all had low postnatal r_c_SO_2_, supporting our findings. Second, multiple testing may have introduced type 1 errors and our findings need confirmation by larger cohorts. Third, behavior and EF were assessed using parental questionnaires, and not by actually testing the children. Final, our cohort consisted of a heterogeneous group of term and preterm infants, possibly including FGR of different entities. Although this cohort excluded children with chromosomal abnormalities and placental histologic findings did not significantly differ between FGR children with and without brain-sparing, placental insufficiency may have been less severe in those without fetal brain-sparing.

In conclusion, in this 4-year-old FGR cohort, fetal brain-sparing and high postnatal r_c_SO_2_ were—as a reflection of one and the same mechanism, but also independently—associated with better behavior and EF. Gestational age at onset of FGR relative to cerebral oxygen demands may play a role. However, high r_c_SO_2_ on day two after birth was also associated with poorer performance IQ, possibly involving intracerebral hemodynamic redistribution upon cardiac decompensation and oxidative stress. Elective delivery based on abnormal DV waveforms indicating decline of cardiac function and protective brain-sparing, together with postnatal measures reducing the cerebral hypo- and hyperoxic burden, may benefit long-term neurodevelopment following FGR. Moreover, FGR infants without fetal brain-sparing seem to require closer follow-up.

## Data Availability Statement

The datasets generated for this study are available on request to the corresponding author.

## Ethics Statement

The studies involving human participants were reviewed and approved by Medical Ethical Committee, University Medical Center Groningen. Written informed consent to participate in this study was provided by the participants' legal guardian/next of kin.

## Author Contributions

AR conceptualized the study, collected, analyzed and interpreted the data and drafted the first manuscript. JT contributed to study design and acquisition of perinatal data and critically revised the manuscript for its intellectual content. SS contributed to acquisition, analysis and interpretation of the presented data and critically revised the manuscript for its intellectual content. AF and AH contributed to acquisition and interpretation of follow-up data and critically revised the manuscript for its intellectual content. MS contributed to analysis and interpretation of placental data and critically revised the manuscript for its intellectual content. CB contributed to study design, collection and interpretation of fetal Doppler data and critically revised the manuscript for its intellectual content. AB, SAS, and EK contributed to study design and interpretation of the data and critically revised the manuscript for its intellectual content. All authors gave final approval of the version to be published and agree to be accountable for all aspects of the work.

## Conflict of Interest

The authors declare that the research was conducted in the absence of any commercial or financial relationships that could be construed as a potential conflict of interest.
